# Examining double standards in layoff preferences and expectations for gender, age, and ethnicity when violating the social norm of vaccination

**DOI:** 10.1038/s41598-023-48829-4

**Published:** 2024-01-02

**Authors:** Cristóbal Moya, Sebastian Sattler, Shannon Taflinger, Carsten Sauer

**Affiliations:** 1 Faculty of Sociology, Bielefeld University, 33615 Bielefeld, Germany; 2 DIW Berlin, 10117 Berlin, Germany; 3 Pragmatic Health Ethics Research Unit, Institut de Recherches Cliniques de Montréal, Montreal, H2W 1R7 Canada; 4 Institute of Sociology and Social Psychology, University of Cologne, 50931 Cologne, Germany

**Keywords:** Psychology, Human behaviour

## Abstract

Whether vaccination refusal is perceived as a social norm violation that affects layoff decisions has not been tested. Also unknown is whether ascribed low-status groups are subject to double standards when they violate norms, experiencing stronger sanctions in layoff preferences and expectations, and whether work performance attenuates such sanctioning. Therefore, we study layoff preferences and expectations using a discrete choice experiment within a large representative online survey in Germany (*N* = 12,136). Respondents chose between two employee profiles, each with information about ascribed characteristics signaling different status groups (gender, age, and ethnicity), work performance (work quality and quantity, and social skills), and whether the employees refused to vaccinate against COVID-19. We found that employees who refused vaccination were more likely to be preferred and expected to be laid off. Respondents also expected double standards regarding layoffs due to vaccination refusal, hence, harsher treatment of females and older employees. Nonetheless, their preferences did not reflect such double standards. We found little support that high work performance attenuates these sanctions and double standards, opening questions about the conditions under which social biases arise. Our results suggest detrimental consequences of vaccination refusal for individuals, the labor market, and acceptance of health policies.

## Introduction

During the early stages of the COVID-19 (SARS-CoV-2) pandemic, the rapid development of vaccines against the virus was deemed a crucial intervention to save lives and counteract the negative effects of the pandemic on social life. However, after the development of vaccines, public health officials subsequently faced the challenge of vaccinating the highest possible share of the population against the COVID-19 virus. To this aim, many countries relied on public health campaigns to inform citizens about the benefits of being vaccinated for both the vaccinated and those with whom they interact. For example, in Germany the government led campaigns aimed to inform and motivate people to get vaccinated^1,2^. In May 2022 (around the time we conducted our study), more than three-quarters of the German population was fully vaccinated. While vaccination rates vary around the globe, in many countries the majority of the population is vaccinated^3^. The generally positive attitudes toward vaccination^4^ as well as the high uptake of the vaccine among the public in Germany and in many other countries suggest that vaccination against COVID-19 has likely become a social norm^5^.

As has occurred with previous vaccination interventions, accepting or refusing vaccination is a contested domain with moral implications that can lead to social biases and discrimination between the vaccinated and the unvaccinated^6,7^. While social research on vaccination against COVID-19 has largely focused on causes of vaccination hesitation and refusal^4,8–10^, recent studies have explored other social consequences of vaccination as a public health intervention. For example, a study conducted in 21 countries showed that vaccinated people held discriminatory attitudes toward unvaccinated individuals and that vaccinated individuals were more likely to view unvaccinated individuals as “untrustworthy” and “incompetent”^11^. Also in the context of online experiments before the COVID-19 pandemic, vaccinated individuals were likely to sanction unvaccinated individuals for not being vaccinated^12^.

Nevertheless, the social consequences of such mass vaccination in work contexts remain relatively underresearched. During the COVID-19 pandemic, many organizations reduced their operations and furloughed or laid off employees due to financial hardship, resulting in an 8% decrease in global working hours in 2020 compared to 2019 (equivalent to 255 million full-time jobs)^13^. Especially in times of economic recession, companies must set criteria for deciding which employees to lay off^14^. As vaccination likely operates as a social norm, refusing to get vaccinated could be considered a consequential criterion for layoff decisions. This proposition is supported by the aforementioned portrayals of unvaccinated individuals as less competent^11^, given that competence in task performance is a legitimate and widely accepted criterion for layoff decisions^15,16^.

While research has identified criteria used by organizations when choosing who to lay off^16^, it is not clear how non-experts behave when confronting such a decision. While supervisors and human resource representatives are generally those who have the decision-making power to lay off employees, examining non-expert preferences and expectations about who to lay off has a threefold relevance. First, assessing the role of vaccination refusal on layoff *preferences,* regarding who *should* be laid off, and layoff *expectations,* of who *will* be laid off, provides evidence of the extent to which vaccination is a social norm whose violation should be sanctioned^17,18^. As future health crises are likely^19^, it is important to understand how people react to the interventions used to tackle such situations. Second, public opinion regarding layoff preferences is informative for expert decision-making processes: extant scholarship evidences that layoffs affect both the laid-off workers and those who survive layoffs^16^. Therefore, layoff decision-makers can reduce the scarring effects for survivors by taking into account non-expert rationales for layoff decisions. Furthermore, a better understanding of layoff rationales based on vaccination refusal can provide insights to design policies that make layoff decisions more procedurally just, which is related to higher compliance and discretionary efforts from workers and less work demotivation within organizations^20^. Third, expectations about social biases and discrimination in layoff decisions, even when in clear contrast with individual preferences, can impact how people behave. As a self-fulfilling prophecy, employees may act *as if* these expectations were real, driving changes at the individual and collective level^21–23^. Therefore, in this study we focus on whether refusing to get vaccinated is a relevant criterion for layoff preferences and expectations of the lay German public, as well as its potential heterogeneity based on ascribed characteristics (gender, age and ethnicity) and achieved characteristics (work performance).

### Layoff preferences and expectations depending on vaccination refusal

Vaccination has been one of the most important interventions to confront COVID-19 in the long term, rapidly adopted by authorities around the world where vaccines were available^3,24^. From an epidemiological perspective, getting vaccinated against the virus not only contributes to individual protection against severe disease outcomes^24,25^, but also adds to the collective protection for those around, such as coworkers and close contacts^26,27^. Hence, vaccination refusal is an individual health decision with consequences beyond oneself. The collective effect of vaccination also entails that unvaccinated individuals can still benefit from being surrounded by vaccinated individuals, thus, vaccination is a public good subject to the free rider problem^28^. A common way in which free-riding behaviors are mitigated is through the development of social norms that sanction such behaviors^29^. Social norms are defined as a rule of behavior based on the combination of beliefs in a *normative expectation* that others believe they ought to conform to the behavior and an *empirical expectation* that most people conform to the behavior^5^. In Germany, there is support for the empirical expectation that others conform to COVID-19 vaccination, as there is a high uptake of the COVID-19 vaccine and positive attitudes toward the vaccine^3,4^.

Moreover, social norms also imply sanctions for norm violations. For example, sanctioning behavior toward those who refuse vaccination has been found in the form of negative attitudes toward the unvaccinated in Germany, among other countries^11^. Building upon the conception of vaccination as a social norm and the sanctioning of its violation, we focus on the consequences of refusing vaccination in work contexts. We study whether and—if so—to which extent refusing vaccination is sanctioned by expressing higher layoff preferences (a higher likelihood of preferring to lay off candidates who refuse vaccination), and if there is an expectation that employers will sanction such behavior (a higher likelihood of expecting such candidates to be laid off by employers). Drawing from the literature on deservingness, it can be argued that non-compliance with vaccination norms can serve as a heuristic to determine who should be considered for layoffs^30^. Therefore, if being vaccinated operates as a social norm, when faced with the decision of laying off two potential candidates, we expect lay people to be more likely to prefer the employee who refuses vaccination over an employee who is vaccinated (*H1a*). Likewise, we hypothesize that laypeople will also expect employers to be more likely to lay off the candidate who refuses to get vaccinated (*H1b*). In an exploratory manner, we also test the potential heterogeneity of these hypotheses based on participants' attitudes toward vaccination. If getting vaccinated operates as a social norm, people with negative attitudes toward vaccination would not be more likely to prefer to lay off an employee who refuses vaccination, but they would still be more likely to expect them to be laid off.

### Double standards for vaccination refusal among status groups

If vaccination refusal is socially sanctioned with higher layoff preferences and expectations, the sanctions might still be heterogeneous depending on who violates the norm of vaccination. Research on double standards evidences how standards in diverse domains, such as competence judgments in work contexts, depend on the status of the individual being evaluated, whereby low-status groups are subject to stricter standards. In simple terms, status groups refer to a comparative social ranking in terms of the social esteem, honor, and respect accorded to them^31^. Double standards are theorized to occur independent of objective performance indicators and disadvantage low-status groups^32–34^. For example, when deciding who should get a promotion among two employees with equal performance indicators (e.g., amount of sales), people are expected to be biased against the low status employee, perceiving them as less competent. Double standards theory follows expectation states theory^35,36^, proposing that performance expectations are derived from status groups, which can be either task-specific or diffuse (i.e., applied to various kinds of tasks). Common examples of diffuse status characteristics are social status groups based on ascribed characteristics, such as gender, age, and ethnicity^31,37^.

Women, older people, and ethnic minorities are subject to discrimination and social biases in the workplace, which arise through different mechanisms^38–42^. Whether due to negative stereotypes that are translated into prejudice in work settings^43^, preferring members of the same social status group^44^, or inferring attributions from the social status of the group^45^, previous research demonstrates biases against women, older people, and ethnic minorities in work contexts. The relevance of these mechanisms differs among these status groups, but the outcomes in terms of disadvantage are similar, e.g., lower chances of being hired and higher chances of being laid off. Disadvantages in hiring are consistently found for some groups based on race and ethnicity^42,46,47^, as well as for older people^48^. This is also the case for women^49^, but also with contrasting findings^50^. While studies on double standards usually examine hiring decisions, double standards theory has also been applied in the case of differential punishment for misconduct depending on gender and ethnicity^44^. Here, we seek to apply it to sanctioning social norm violations via layoff preferences and expectations in a public health crisis.

According to double standards theory^33^, members of high-status groups are generally perceived as more competent in domains such as work, hence, they are *expected* to perform well with regard to specific performance evaluations, while the contrary holds for members of low-status groups. As a consequence, ascribed characteristics indicating belonging to low social status groups can lead to stricter standards for layoffs. In this line, if vaccination refusal is sanctioned with layoffs, we expect women, older people, and ethnic minorities to be subject to stricter sanctions when refusing to vaccinate compared to men, younger people, and members of the ethnic majority. Hence, we expect the positive effect of refusing to get vaccinated on being preferred (*H2a*) and expected (*H2b*) to be laid off to be stronger for women, older people, and ethnic minorities.

### Double standards for vaccination refusal and work performance

Work performance is a widely used criterion within organizations for assessing employees and making important decisions, such as layoffs^15,16^. The evaluation of workers' performance within organizations operates as a legitimate criterion for laying off employees, that is commonly accepted both by legal authorities and employees^15,51^. As a socially valid criterion, people may rely on it when confronted with choosing who should be laid off and will expect employers to do so as well. In addition, from an organizational perspective, employees’ performance is rewarded due to its benefit to organizations, and the rewards for performance act as an exchange relationship that motivates workers^52,53^. Given that work performance appears as a legitimate and rational criterion to lay off employees, we expect that candidates who show a lower performance will be preferred to be laid off.

Considering the pivotal role of work performance for layoff decisions and the potential for vaccination refusal to function as a heuristic, we hypothesize that high work performance (e.g., work quality, work quantity, and social skills) will attenuate the positive effect of vaccination refusal on layoff preferences and expectations. First, people may seek to mitigate losses in human capital in work organizations by preferring to lay off people with lower work performance. Thus, the norm violation of vaccination refusal might be weighed against these costs of human capital and could lead to attenuating effects of higher work performance. Therefore, we expect high work performance to attenuate the proposed higher likelihood of being preferred and expected to be laid off among employees who refuse vaccination. In other words, when information about performance evaluation shows a higher performance, vaccination refusal is expected to have a weaker positive effect on layoff preferences (*H3a*). Similarly, people may expect that performance will also orient employers' decisions when choosing who to lay off among candidates who refuse or comply with getting vaccinated. Thus, we also postulate that people expect employers to consider vaccination refusal as less consequential when workers' performance is higher (*H3b*).

### Double standards for vaccination refusal and work performance among status groups

Double standards theory claims that members of low-status groups are subject to stricter standards compared to high-status groups, so that in the event of equivalent performance by members from low- and high-status groups, the latter are more likely to be judged to have met the performance standard^32–34^. Nevertheless, work performance is a socially valid criterion in layoff decisions and is thus expected to attenuate the effect of norm violations, such as vaccination refusal. We posit that the attenuating role of high performance on the effect of vaccination refusal on layoff preferences and expectations is less relevant for low-status groups (women, older people, and ethnic minorities) compared to high-status groups (men, younger people, and ethnic majority). In other words, low-status groups receive fewer benefits in terms of the attenuating role of high performance in the effect of vaccination refusal on layoff preferences (*H4a*) and expectations (*H4b*).

### The current study

We employed a discrete choice experiment to assess people's preferences and expectations for laying off employees who refuse to get vaccinated against COVID-19. Our study was conducted using a representative sample of the adult population in Germany at a time when several governments around the world, including Germany, either discussed or decided to make vaccination mandatory for members of particular age groups or occupations, such as healthcare workers, oftentimes due to increasing infection rates as a result of the Delta and Omicron variant and because of a slowdown in vaccination rates^54,55^. This led to tense political and public discussions, protests^56–58^, and the Federal Anti-Discrimination Agency in Germany to clarify that vaccination status is not a protected class under the German General Equal Treatment Act^59^. Moreover, the German Federal Labour Court ruled the termination of employment due to vaccination status can be lawful under certain circumstances^60–62^. In light of the high vaccination rates and positive attitudes toward vaccination in Germany, we hypothesized that individuals who do not follow this social norm will be sanctioned by being both preferred and expected to be laid off. In line with double standards theory^32,33^, we posited that this positive effect will be stronger for low-status groups based on ascribed characteristics, i.e., women, older people, and ethnic minorities will be subject to stricter standards when refusing to get vaccinated. We then assessed whether higher work performance—in terms of three key indicators typically considered in real-world performance evaluations (i.e., work quality, work quantity, and social skills)—can attenuate the positive effect of vaccination refusal on layoff preferences and expectations. Finally, we examined whether the standards for maintaining one’s job are higher for members of low-status groups depending on their work performance. The main study hypotheses are summarized in Table S1 in the Supplements. By extending the focus of double standards to sanctioning social norm violations in the context of COVID-19, we seek to contribute to the literature on discrimination and social biases in the labor market; attitudes during public health crises; and public reactions toward policies.

## Methods

### Design and participants

We conducted a web-based study with a representative sample of participants living in Germany. In order to recruit participants, we used the survey-provider *bilendi & respondi*, which provides an actively managed panel with voluntary participation and a double opt-in registration process, including permanent quality control. We used a quota sample representative of the German population with regard to gender, age (18–75), education, and federal state. 14,651 participants started the survey, of whom 14,205 provided informed consent, 13,027 completed the survey, and 12,183 remained after data cleaning. Data collection occurred between April 19–May 5, 2022. Respondents who completed the study received a small monetary compensation (€1). After removing respondents with missing values on all variables required for the analyses, 12,136 respondents remain in the analytical sample (51.0% females and 0.2% diverse gender; *mean* age = 46.5, *standard deviation* = 15.9). Approximately 5.0% (602 of 12,087) of the respondents indicated that they were born outside of Germany, and 14.0% (1677 of 12,089) were born abroad or had a parent born outside of Germany (1.4% of the 12,136 respondents did not answer all ethnicity questions).

### Experimental setup

In order to understand how employee characteristics influence the layoff decisions made by laypeople, our study employs a discrete choice experiment^45,63–65^. Thereby, participants were informed that a fictional medium-sized company must lay off one employee and were shown the human resources profiles of two potential candidates to be laid off (see Table 1). The human resources profiles included nine experimentally varied pieces of information for each candidate of which one was their COVID-19 vaccination status (fully vaccinated or refused vaccination). During the pandemic, employers were allowed and also obliged (according to the Infection Protection Act) to check the 3G status of employees (i.e., whether they were tested, vaccinated, or recovered) when deciding who could attend work in person and/or access an office or other facilities. Thus, employers could ask employees to report their vaccination status. Moreover, candidates’ profiles included their names, which conveyed information regarding two status group indicators, gender (male or female) and ethnicity (German or Turkish ethnic background). The male names “Christian Wagner” and “Michael Berger” and the female names “Susanne Wagner” and “Kathrin Schneider” indicated a German background, while the male names “Mehmet Yilmaz” and “Mustafa Yücel” and the female names “Ayşe Yilmaz” and “Fatma Demir” indicated a Turkish background^cf.^^66,67^. As a further status group indicator, information on the candidates’ ages was provided (35 or 55 years old). We used three indicators for task performance, namely work quantity, work quality and social skills, since they are important criteria in the assessment of an employee’s achievements and capacity^68^ and they can be seen as important determinants of layoff chances^15^. Also, tenure in the company and nepotism (i.e., whether there are family members working in the company) were described, but due to the focus of this manuscript on the intersection of ascribed status characteristics and work performance using double standards theory, duration in the company and nepotism were not included in the analyses. In future papers with a different focus than the present study, we want to, for example, examine in greater detail the pattern of ageism and ethnic discrimination in which duration in the company and nepotism can be protective or risk factors for layoff preferences and expectations. By assessing these two further factors within the same large experiment, we wanted to reduce survey costs and also the burden for participants. Considering all experimental factors had no implications for the quantities of interest of this study. The results with these factors are available in the Supplements (Tables S9 and S10). Given the orthogonality of the experimental design, the exclusions do not affect the results.

**Table 1:**
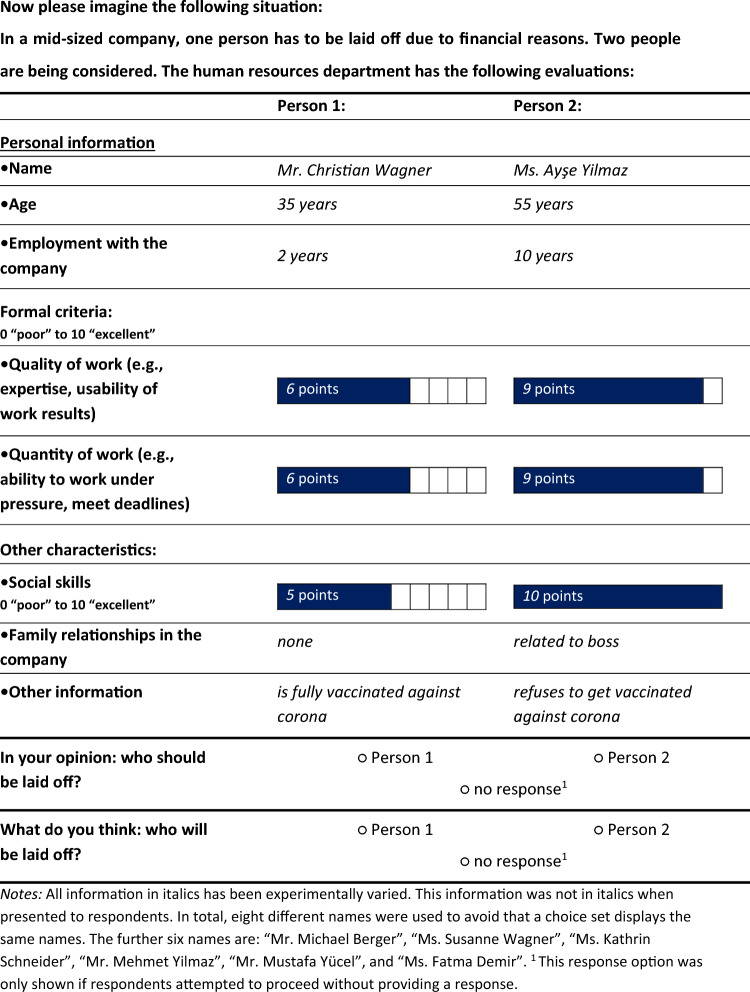
Discrete choice experiment showing one exemplary choice set representing all different alternatives.

All information in italics has been experimentally varied. This information was not in italics when presented to respondents. In total, eight different names were used to avoid that a choice set displays the same names. The further six names are: “Mr. Michael Berger”, “Ms. Susanne Wagner”, “Ms. Kathrin Schneider”, “Mr. Mehmet Yilmaz”, “Mr. Mustafa Yücel”, and “Ms. Fatma Demir”.^1^ This response option was only shown if respondents attempted to proceed without providing a response.

Each piece of information in the profiles was experimentally manipulated for each of the employees yielding a 2 × (2^9^) design, resulting in a total of 512 possible combinations per displayed person and 1024 choice sets. For our study, we selected 150 choice sets for both persons via *D*-efficient sampling^69^ using the software SAS 9.4. Thereby, an algorithm specified a sample characterized by minimal correlation between the dimensions and maximal variance and balance of the frequency of the vignette levels. We assigned each participant to one choice set and each choice set was rated multiple times (*mean* = 81.2; *range* = 74–87) by different participants. After reading the vignette, participants were asked about their layoff preferences and expectations.

### Measures

#### Layoff preference

Respondents were asked about their layoff preference, i.e., which of the two candidates, in their opinion, should be laid off (see Table 1). If respondents attempted to proceed without a response, the additional response option “no response” was shown (chosen by *N* = 41; 0.3%).

#### Layoff expectation

We also assessed their layoff expectation, i.e., which of the two candidates they expect will be laid off (see Table 1). Again, respondents could additionally choose “no response”, after first attempting to proceed without providing a response (chosen by *N* = 17; 0.1%). The order of these two questions was experimentally manipulated and the order did not have an effect on either layoff preferences (*Odds Ratio* (*OR*) = 1.00, *p* = 1.00) or expectations (*OR* = 1.00, *p* = 1.00) (see Supplement Tables S11 and S12).

#### Attitude toward COVID-19 vaccination

Respondents answered the question, “What is your opinion about the Corona vaccination? I am…” with a five-point scale from “Strongly in favor” [value 1], “Rather in favor” [2], “Undecided” [3], “Rather against” [4], “Strongly against” [5] (*mean* = 1.92, *standard deviation* = 1.32, *median* = 1.00).

### Pretests

To examine the comprehensibility of the instructions and choice sets, we conducted cognitive pretests (*N* = 8) with the think-aloud technique and probing questions^70^ as well as a quantitative pretest (*N* = 85). Results of both pretests demonstrated that the materials were suitable for the main study.

### Statistical analysis

We used mixed logit models^71^ to examine participants’ preferences and expectations for the two candidates in the choice set of the experiment. Intercepts were considered as random parameters to account for the nesting of the possible candidates within respondents, i.e., each respondent evaluated two candidates. The seven focal experimental factors were used as predictors and, following the study hypotheses, parameterized as main effects and two- or three-way interactions (see Supplement Tables S2–S8). Due to multiple hypothesis testing, we use the false discovery rate method (controlling the expected proportion of falsely rejected hypotheses) to adjust the *p*-values^72^.

Because all experimental factors were dichotomous, we report average contrasts as differences in predicted average probabilities between both levels of the experimental factors. For example, an average probability difference of 0.1 between candidates refusing to vaccinate and those fully vaccinated for layoff preferences means that, on average, respondents are more likely to prefer to layoff those who refuse vaccination by ten percentage points compared to those fully vaccinated. To assess potential differences in the effect of vaccination refusal by candidates’ ascribed characteristics and work performance indicators, we used difference-in-differences and difference-in-differences-in-differences^73^. For example, the difference-in-differences of gender tests the difference in the average contrasts of vaccination refusal between female and male candidates. So, for example, a difference-in-differences of refusing to vaccinate between female and male candidates of 0.05 for layoff preference would mean that the average difference in the probability of being preferred to be laid off when refusing to vaccinate is five percentage points higher for women compared to men. The difference-in-differences-in-differences tests the difference between high and low work performance (e.g., work quality) from the aforementioned differences. The main results are based on Model 4a (Table S7) and Model 4b (Table S8). The average effects of the experimental factors are reported in Table S2, while Table S3 shows that these results hold with a simpler model parameterization (Models 1a and 1b from Tables S7 and S8, respectively). All analyses were conducted using R 4.3.0^74^.

### Ethics approval

Ethics approval was received from the Faculty of Management, Economics, and Social Sciences of the University of Cologne (ethics approval numbers: 220013DM). Our work also aligns with the Code of Ethics of the American Sociological Association (ASA) and although our study is not a medical study, we adhere to the Code of Ethics of the World Medical Association (Declaration of Helsinki) to protect human research participants.

### Consent to participate

Informed consent was obtained from all study participants.

## Results

### Layoff preferences and expectations depending on vaccination refusal

Candidates who refused to get vaccinated had a higher likelihood of being preferred to be laid off compared to candidates who were fully vaccinated (on average, 22 percentage points, *p* < 0.001), supporting *H1a*. Likewise, candidates who refused vaccination had a higher likelihood of being expected to be laid off (on average, 20 percentage points, *p* < 0.001), supporting *H1b*. See Table S2 for an overview of the study findings.

**Figure 1:**
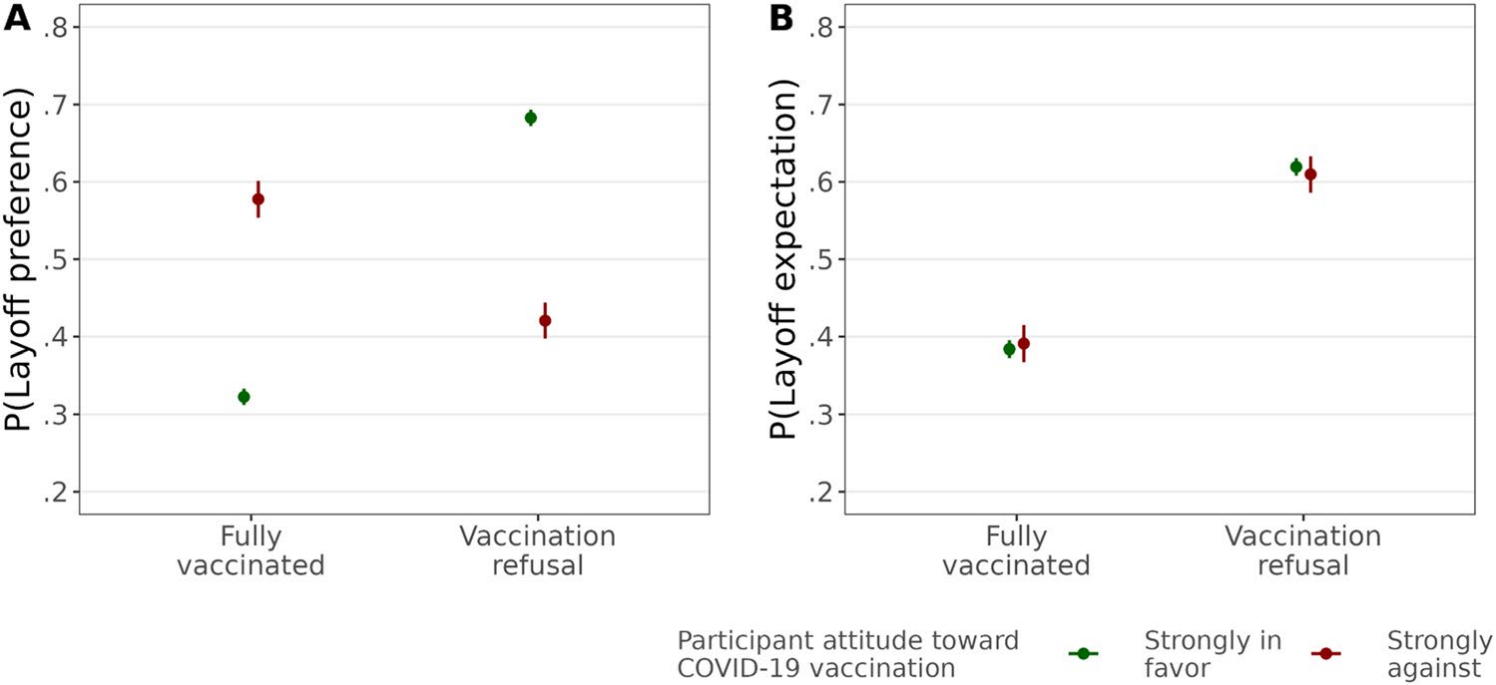
Effect of vaccination refusal on layoff preferences and expectations by participants’ attitudes toward vaccination. Average predicted probabilities of layoff preferences (**A**) and layoff expectations (**B**) from interactions between the candidate’s vaccination status and the participant’s attitude toward vaccination. Ribbons represent 95%-confidence intervals (estimations based on Models 5a and 5b in Tables S7 and S8, *N* = 12,029 individuals).

Despite these similar average contrasts of vaccination refusal between layoff preferences and expectations, the correlation between both was positive, but rather weak among the choice sets of candidates (*r*(12,134) = 0.22, *p* < 0.001). Moreover, we observed considerable heterogeneity in the effect of vaccination refusal on layoff preferences depending on the participant’s attitude toward vaccination: the more negative the attitude, the less likely they prefer to lay off candidates refusing vaccination, and the more positive the attitude, the more likely they voiced such a preference (Fig. 1A). The effect of vaccination refusal on layoff expectations did not vary by participants’ attitudes toward vaccination (Panel B).

### Double standards for vaccination refusal depending on ascribed status group

**Figure 2:**
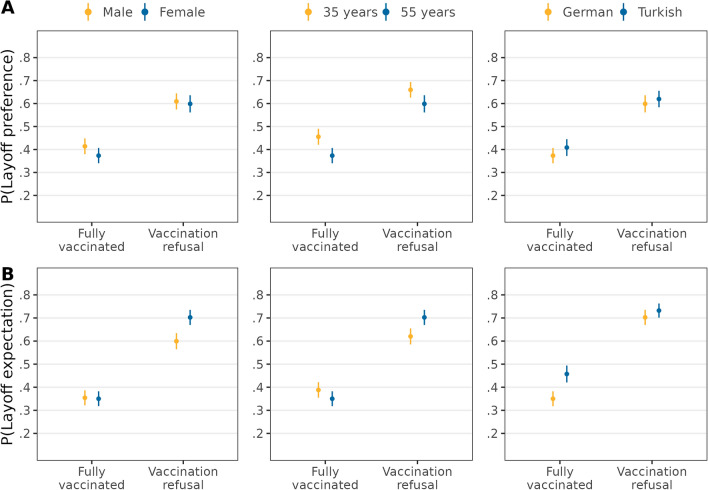
Double standards for vaccination refusal depending on ascribed status group. Average predicted probabilities (with 95%-confidence intervals) of layoff preferences (**A**) and layoff expectations (**B**) from interactions between vaccination status and ascribed status group characteristics (estimations based on Models 4a and 4b in Tables S7 and S8, *N* = 12,136 individuals).

In contrast to *H2a*, candidates from low-status groups (women, older people, and ethnic minorities)—as compared to high-status groups (men, younger people, and ethnic majority)—who refused to get vaccinated were not more likely to be preferred to be laid off (see Fig. 2 and Table S4). In line with *H2b*, candidates refusing vaccination were more likely to be expected to be laid off when they were women (∆ = 0.11, *95%-CI* = [0.06,0.15]) or older (∆ = 0.12, *95%-CI* = [0.07,0.17]), whereby women and older employees were 11 and 12 percentage points more likely to be expected to be laid off, respectively. Conversely, candidates with Turkish ethnicity had a higher likelihood of being expected to be laid off compared to German candidates when they were fully vaccinated, but they were equally sanctioned when refusing vaccination (∆ = − 0.08, *95%-CI* = [− 0.12,− 0.03]). Thus, *H2b* showed partial support.

### Double standards for vaccination refusal depending on work performance

**Figure 3:**
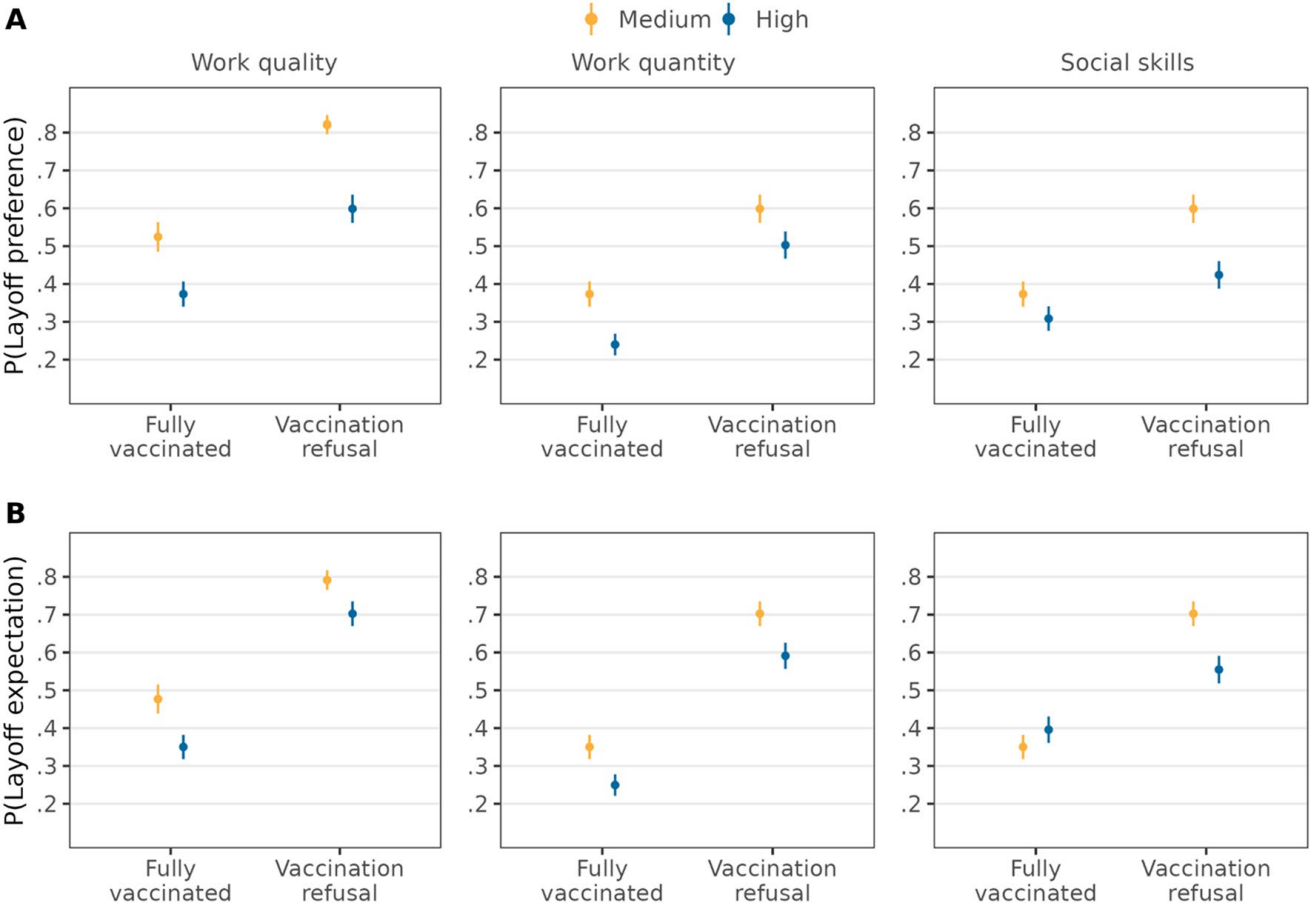
Double standards for vaccination refusal depending on work performance. Average predicted probabilities (with 95%-confidence intervals) of layoff preferences (**A**) and layoff expectations (**B**) from interactions between vaccination status and candidates’ performance (estimations based on Models 4a and 4b in Tables S7 and S8, *N* = 12,136 individuals).

The higher likelihood of being preferred to be laid off among candidates who refuse vaccination was attenuated for candidates showing a high performance in terms of work quality (∆ = − 0.07, *95%-CI* = [− 0.12,− 0.02]) and social skills (∆ = − 0.11, *95%-CI* = [− 0.16,− 0.06]) (see Fig. 3 and Table S5). We did not find such an attenuation for candidates with high performance in terms of work quantity (∆ = 0.04, *95%-CI* = [− 0.01,0.09]). This suggests partial support for *H3a*. The higher probability of being expected to be laid off due to vaccination refusal was reduced only for candidates with high social skills (∆ = − 0.19, *95%-CI* = [− 0.24,− 0.14]). The effect of vaccination refusal on layoff expectations did not vary by levels of the other two indicators of work performance. Thus, evidence for *H3b* was also partial.

### Doubled standards for vaccination refusal depending on ascribed status group and work performance

**Figure 4:**
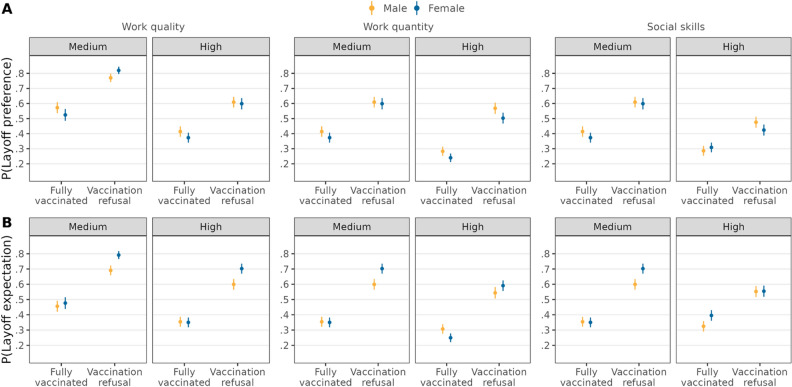
Double standards for vaccination refusal depending on gender and work performance. Average predicted probabilities (with 95%-confidence intervals) of layoff preferences (**A**) and layoff expectations (**B**) from interactions between vaccination status, gender, and work performance (estimations based on Models 4a and 4b in Tables S7 and S8, *N* = 12,136 individuals).

Moreover, the positive effect of refusing vaccination on being preferred to be laid off was attenuated to a greater extent for female, compared to male, candidates by work quality (∆ = − 0.07, *95%-CI* = [− 0.12,− 0.02]) and social skills (∆ = − 0.10, *95%-CI* = [− 0.16,− 0.05]). More specifically, the positive effect of refusing vaccination on being preferred to be laid off was attenuated for women, but not for men (Fig. 4, Table S6). In the case of layoff expectations, the positive effect of refusing vaccination was attenuated by social skills to a larger extent for female, compared to male, candidates (∆ = − 0.18, *95%-CI* = [− 0.23,− 0.13]). Similar to layoff preferences, the positive effect of vaccination refusal on being expected to be laid off did not vary for men by social skills. No statistically significant differences were observed for work quantity (layoff preferences) or for work quantity and work quality (layoff expectations). Overall, results do not support *H4a* and *H4b* for gender.

**Figure 5:**
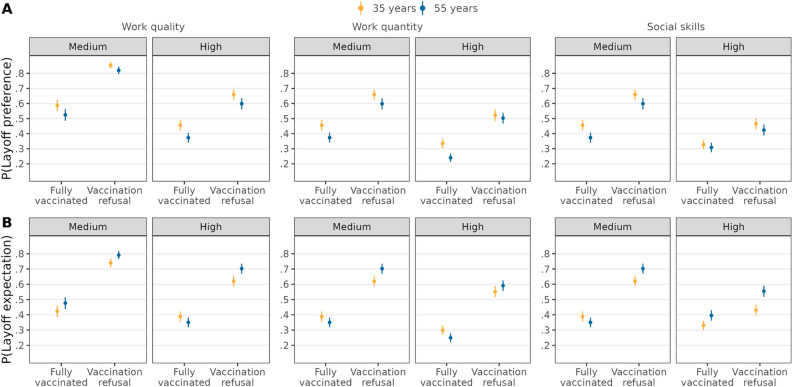
Double standards for vaccination refusal depending on age and work performance. Average predicted probabilities (with 95%-confidence intervals) of layoff preferences (**A**) and layoff expectations (**B**) from interactions between vaccination status, age, and work performance (estimations based on Models 4a and 4b in Tables S7 and S8, *N* = 12,136 individuals).

There were no statistically significant differences in the effect of vaccination refusal on layoff preferences among candidates of different ages and levels of work performance (Fig. 5, Table S6). Thus, there is no support for *H4a* for age. The positive effect of vaccination refusal on layoff expectations was attenuated for younger candidates when work quality was high. However, high work quality actually intensified this effect for older candidates. Therefore, supporting *H4b*, the positive effect of refusing vaccination on layoff expectations was augmented for older candidates, compared to younger ones (∆ = 0.12, *95%-CI* = [0.08,0.17]). Both older and younger candidates showed an attenuation of the positive effect of vaccination refusal on being expected to be laid off by high performance in social skills. Nonetheless, against *H4b*, the positive effect of refusing vaccination on layoff expectations was attenuated to a higher extent for older, compared to younger, candidates (∆ = − 0.06, *95%-CI* = [− 0.11,− 0.01]). There was not a statistically significant difference in the effect of vaccination refusal on layoff expectations among candidates of different ages and levels of work quantity. Hence, there is a lack of support for *H4a* and *H4b* for age.

**Figure 6:**
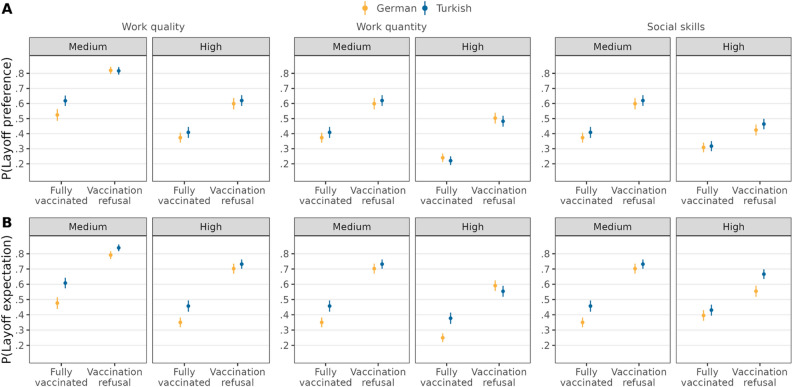
Double standards for vaccination refusal depending on ethnicity and work performance. Average predicted probabilities (with 95%-confidence intervals) of layoff preferences (**A**) and layoff expectations (**B**) from interactions between vaccination status, ethnicity, and work performance (estimations based on Models 4a and 4b in Tables S7 and S8, *N* = 12,136 individuals).

The positive effect of vaccination refusal on being preferred to be laid off was attenuated among candidates of German ethnicity who displayed high work quality, while there was no difference among candidates of Turkish ethnicity (∆ = 0.08, *95%-CI* = [0.03,0.13]). Both work quantity and social skills did not lead to statistically significant differences in the effect of vaccination refusal on layoff preferences by ethnicity (Fig. 6, Table S7). Thus, only one out of three indicators support *H4a* for ethnicity. The positive effect of vaccination refusal on being expected to be laid off was attenuated for both Turkish and German ethnic candidates with high performance in work quantity and social skills. However, the positive effect of refusing vaccination on layoff expectations was attenuated to a higher extent for candidates of Turkish ethnicity, compared to those of German ethnicity, when work quantity was high (∆ = − 0.09, *95%-CI* = [− 0.14,− 0.04]). Conversely, the positive effect of refusing vaccination on layoff expectations was attenuated to a lower extent for Turkish ethnic, compared to German ethnic candidates, when social skills were high (∆ = 0.15, *95%-CI* = [0.11,− 0.20]). There was no statistically significant difference in the effect of vaccination refusal on layoff expectations among candidates of Turkish and German ethnicities and different levels of work quality. Thus, the evidence of *H4b* for ethnicity is mixed with results showing support, no support, or opposite evidence.

## Discussion

This study contributes to understanding how social norm violations in a public health crisis are consequential for work contexts and how they can subject low-status groups to social biases, especially in terms of layoff expectations. Using a large-scale discrete choice experiment, this study examined potential social biases in people's preferences and expectations regarding laying off employees who refuse to get vaccinated against COVID-19. First, we showed how vaccination appears as a social norm whose violation entails a sanction for employees: candidates refusing vaccination were 22 percentage points more likely to be preferred to be laid off and 20 percentage points more likely to be expected to be laid off by the employer (supporting *H1a* and *H1b*). We also find strong evidence of vaccination as a social norm stemming from respondents’ layoff preferences and expectations: among participants with negative attitudes toward vaccination, refusing vaccination decreased the likelihood of being preferred to be laid off, while the effect of vaccination status on layoff expectations did not differ between participants with positive and negative attitudes. This signals that participants have the expectation that others view vaccination as a socially desired behavior and that violations of this norm will be subject to sanctions, despite their own preferences in favor or against vaccination. Our findings support the idea of vaccination as a social norm as well as align with previous studies showing discriminatory attitudes toward non-vaccinated individuals^7,11,12^.

Second, our study tested claims from double standards theory that ascribed status characteristics signal competence, and as a result, individuals of high-status groups are subject to more lenient standards than low-status groups^32–34^. We provided partial evidence of double standards concerning the sanctioning of vaccination refusal depending on ascribed status characteristics. On the one hand, people use the same standards for different status groups when evaluating who was preferred to be laid off due to vaccination refusal (against *H2a*). While such a finding can be seen as generally normatively positive regarding the lack of discrimination, we also recognize that individuals may not be willing to voice discriminatory preferences (see “Limitations and directions for future research” below). On the other hand, we found evidence of double standards regarding which candidate they expect to be laid off. Participants expected female and older candidates who refused vaccination to be more likely to be laid off (partly supporting *H2b*). The effects on expectations were quite substantial as women were 11 percentage points and older employees were 12 percentage points more likely to be expected to be laid off when refusing to vaccinate. Interestingly, in the case of candidates of Turkish ethnicity, participants expected stricter standards (compared to German ethnic candidates) when they were fully vaccinated, but they were expected to be equally sanctioned when refusing to vaccinate. Thus, our results do not support the existence of a more direct bias in preferences to sanction social norm violations through layoffs depending on status groups among the lay public. However, the lay public expected employers to subject low-status groups to stricter standards when downsizing during the COVID-19 pandemic. Furthermore, the different direction of the double standards for women and older people, compared to candidates of Turkish ethnicity, suggests that expected social biases against these groups possibly stem from disparate mechanisms. For instance, one noticeable difference is that Turkish people in Germany are a minority, whereas women and older individuals are not. The prevalence of ascribed status groups might be key to explaining different social biases^75^. Future research should examine the concrete mechanisms by which biases in expected sanctions for different ascribed status groups arise.

We found partial evidence on the attenuating impact of heightened work performance on both layoff preferences (*H3a*) and expectations (*H3b*) regarding refusing vaccination. This attenuation was evident in relation to two out of the three performance indicators, work quality and social skills, for layoff preferences, and one indicator, social skills, for layoff expectations. When work quality and social skills were high, vaccination refusal played a less consequential role in layoff preferences; the positive effect of vaccination refusal on layoff preference was 7 and 11 percentage points lower for employees with high performance (compared to medium performance) in these dimensions, respectively. The moderating effect of work performance on vaccination refusal was less clear for layoff expectations since only higher social skills diminished the probability that vaccination refusers were expected to be laid off (19 percentage points lower for candidates with high performance). However, this single effect was relatively strong. Generally, these results show that the sanctioning of vaccination refusal is partially counterweighted by work performance, as the lay public appears to value the human capital contribution of employees to organizations. At the same time, they show that they do not expect companies to give particular attention to high performance in terms of the quality and quantity of work when a social norm is broken.

We also examined whether work performance attenuates double standards concerning the divergent effect of vaccination refusal on layoff preferences and expectations depending on the ascribed status group. Overall, we find little support for the assumption that low-status groups receive a reduced benefit—compared to high-status groups—in terms of high work performance attenuating the higher layoff preferences (*H4a*) and expectations (*H4b*) due to vaccination refusal. In addition to supporting evidence, we often found results opposing our hypotheses (i.e., low-status groups received higher benefits), or no statistically significant differences. The supporting evidence was only found for ethnicity, whereby one work performance indicator showed a stronger attenuation for the high-status group in line with *H4a*. Similarly for *H4b*, the attenuation occurred with one work performance indicator both for ethnicity and age. These results call for rethinking how the double standards for low-status groups can be counteracted by job performance, and signal the need for a deeper understanding of the conditions under which particular performance indicators play a role.

Moreover, the examined layoff preferences and expectations have further implications for the work context and labor markets. Mechanisms such as the self-fulfilling prophecy suggest that people may adjust their behavior and ideas to these expectations, even if they do not share them^22,23^. For example, colleagues might avoid collaborating with members from low-status groups if they expect them to be more exposed to layoffs, due to forming arbitrary or false beliefs, such as attributions of lower competence^23^. Differential expectations about biases against low-status groups in layoff processes have implications in terms of scarring effects for layoff survivors. Survivors might interpret the layoff of selected social groups as confirmation of their expectations, irrespective of whether ascribed characteristics played a role in the decision. Furthermore, low-status group members who perceive their risk of being laid off as higher could increase their work effort with the hope of avoiding being laid off, which may increase stress and exhaustion^53^, adding to the already disparate effects of the pandemic on women, older individuals, and ethnic minorities^76,77^.

### Limitations and directions for future research

Although our study provides a first test of double standards theory in the influence of vaccination refusal on preferences and expectations for laying off employees, it is subject to limitations. Firstly, asking the same participant about their preferences and expectations begs the question of whether this framing evoked the notions of *ought* vs *actual*, activating their identity as the kind of person who does not discriminate against others^78^. In case of discriminatory or morally debatable attitudes or behaviors, respondents might see themselves as morally superior due to a positive illusion and self-enhancement effect^79^. Thus, we should be cautious in interpreting our findings as a lack of double standards in layoff preferences. Also, in line with evidence on more direct discrimination patterns arising in field experiments, rather than survey experiments^80,81^, it would be a contribution to further test double standards with the former. Given research showing that treatment effects found in vignette studies can be also replicated with other designs^82,83^, we further encourage the use of other designs as an avenue for future research. Moreover, we tested specific values of the underlying factors in the candidate profiles. Future research should test further expressions of these values (e.g., diverse gender, different ages, work performance levels) to increase the understanding of the examined processes. In addition, it should be considered that our results reflect one specific state of the COVID-19 pandemic, potentially influenced by the availability of effective vaccines, the spread of the virus, as well as the implementation of other effective measures. For example, our study was conducted during a time in which a potential vaccine mandate was discussed by the German parliament and media, hence, a vaccine mandate was salient and potentially feasible. In contexts with distinct norms regarding vaccination, sanctioning preferences and expectations may also vary. While generally, Germany seems comparable to some countries (e.g., the United States or the United Kingdom) with regard to the level of vaccination hesitancy and acceptance, it also differs from others in which vaccination hesitancy and non-acceptance are higher (e.g., Poland and South Africa) or lower (e.g., Spain and South Korea)^84,85^. Hence, cross-cultural comparisons would be informative to understand to which extent our results are context-specific. While this study focused on COVID-19 vaccination as a social norm during a time in which specific work-related public health policies were discussed and enforced (including vaccination mandates) in many countries^54,55,85^, future research may also focus on differential sanctioning preferences and expectations in the case of other social norms, contexts, and regulations (e.g., future pandemics, the requirement of measles vaccination which currently exists in several countries, or double standards for layoffs in terms of ascriptive status characteristics during economic recessions or firing due to workplace violations)^44^. Also, our large quota-representative sample contributes to the external validity, however, future research should aim to use probability samples. Finally, studies should also investigate whether the observed pattern in layoff preferences and expectations are similar or different for managers or people in human resources departments as compared to the views expressed by the general public, which could further increase external validity of the findings.

## Conclusion

In sum, this study reveals that COVID-19 vaccination operates as a social norm, whose refusal in the work context increases the likelihood of being preferred and expected to be laid off. Thus, our results suggest a willingness to and expectation of sanctioning vaccination refusal. Such sanctioning may be one way to mitigate free-riding (i.e., not risking side effects from vaccination while benefiting from vaccinated others) to achieve a public good in the context of a health crisis^28,29^ and as such, may motivate people to get vaccinated^86,87^. Moreover, sanctioning seems indirectly subject to double standards, namely that some low-status groups are expected to be treated more harshly when they refuse to vaccinate. The expectations of such biases can have relevant implications for labor markets as they may reinforce their already existing disadvantage in layoff processes^88^. We also found that performance is a socially valid criterion for layoffs and partially compensates for the violation of a social norm. Furthermore, there was scarce support for the assumption that the attenuating effect of high work performance on the higher layoff preferences and expectations due to vaccination refusal was lower for low status individuals. These results call for rethinking how the double standards for low-status groups can be counteracted by job performance, and signal the need for a deeper understanding of the conditions under which particular performance indicators play a role. Given the tendencies of expected double standards in layoff processes, our results suggest that employees and organizations could benefit from having transparent and clearly informed criteria during layoffs.

### Supplementary Information

Supplementary Tables.

## Data Availability

The data and computer code analyzed during the current study are available in an Open Science Framework (OSF) repository, 10.17605/OSF.IO/HVG3D.
