# Supplementary material for: Examining double standards in layoff preferences and expectations for gender, age, and ethnicity when violating the social norm of vaccination
Source: Sci Rep. 2024 Jan 2;14:39. doi: 10.1038/s41598-023-48829-4 (PMC10762145; doi:10.1038/s41598-023-48829-4)
Supplement: Supplementary file 1 — Supplementary Tables. [file 41598_2023_48829_MOESM1_ESM.docx]

**Table S1: Study hypotheses and their levels of support.**

| **Hypothesis (H)** | | **Level of support** |
| --- | --- | --- |
| *H1* | Employees who refuse COVID-19 vaccination have a higher probability of being preferred (H1a) and expected (H1b) to be laid off as compared to employees fully vaccinated against COVID-19. | ● Full support for *H1a* ● Full support for *H1b* |
| *H2* | The increase in the probability of being preferred (*H2a*) and expected (*H2b*) to be laid off due to COVID-19 vaccination refusal is higher for low status groups (women, older people, and ethnic minorities) as compared to high status groups (men, younger people, and majority ethnic population). | ● No support for *H2a* ● Partial support for *H2b* (2 out 3 tests show support) |
| *H3* | The increase in the probability of being preferred (*H3a*) and expected (*H3b*) to be laid off due to COVID-19 vaccination refusal is lower for high performing employees as compared to employees with a medium performance level (regarding work quality, work quantity, and social skills). | ● Partial support for *H3a* (2 of 3 tests show support) ● Partial support for *H3b* (1 of 3 tests shows support) |
| *H4* | The increase in the probability of being preferred (*H4a*) and expected (*H4b*) to be laid off due to COVID-19 vaccination refusal is smaller for low status groups (women, older people) and older people as compared to employees fully vaccinated against COVID-19) with higher performance levels (regarding work quality, work quantity, and social skills). | ● Scarce support for *H4a* (1 of 9 tests shows support) ● Scarce support for *H4b* (2 of 9 tests show support) |

**Table S2: Average contrasts of experimental factors for layoff preferences and expectations (*N*=12,136 individuals).**

|  |  |  |  | *95%-CI* | |
| --- | --- | --- | --- | --- | --- |
| Factor | Contrast | *Δ P(.)* | *p* | Lower | Upper |
| **Layoff preferences** | |  |  |  |  |
| Vaccination | Vaccination refusal - Fully vaccinated | 0.22 | <0.001 | 0.21 | 0.23 |
| Gender | Female - Male | -0.02 | 0.006 | -0.03 | -0.01 |
| Age | 55 years -⁠ 35 years | -0.04 | <0.001 | -0.05 | -0.03 |
| Ethnicity | Turkish - German | 0.01 | 0.132 | 0.00 | 0.02 |
| Work quality | High ⁠- Medium | -0.18 | <0.001 | -0.19 | -0.17 |
| Work quantity | High ⁠- Medium | -0.13 | <0.001 | -0.14 | -0.11 |
| Social skills | High ⁠- Medium | -0.14 | <0.001 | -0.15 | -0.13 |
| **Layoff expectations** | |  |  |  |  |
| Vaccination | Vaccination refusal - Fully vaccinated | 0.20 | <0.001 | 0.19 | 0.22 |
| Gender | Female - Male | 0.02 | 0.001 | 0.01 | 0.03 |
| Age | 55 years -⁠ 35 years | 0.06 | <0.001 | 0.04 | 0.07 |
| Ethnicity | Turkish - German | 0.08 | <0.001 | 0.06 | 0.09 |
| Work quality | High ⁠- Medium | -0.10 | <0.001 | -0.12 | -0.09 |
| Work quantity | High ⁠- Medium | -0.07 | <0.001 | -0.08 | -0.06 |
| Social skills | High ⁠- Medium | -0.08 | <0.001 | -0.09 | -0.06 |

***Notes:*** *Δ P(.)* represents the average contrast between levels of experimental factors, i.e., the difference in the predicted average probabilities between both levels of experimental factors. *p* is the *p-*value adjusted by the false discovery rate. *95%-CI* represents 95% confidence interval. Estimates from Models 4a and 4b in Supplement in Tables S7 and S8.

**Table S3: Average contrasts of experimental factors for layoff preferences and expectations (*N*=12,136 individuals).**

|  | | | | *95%-CI* | |
| --- | --- | --- | --- | --- | --- |
| Factor | Contrast | *Δ P(.)* | *p* | Lower | Upper |
| **Layoff preferences** | |  |  |  |  |
| Vaccination | Vaccination refusal - Fully vaccinated | 0.22 | <0.001 | 0.21 | 0.23 |
| Gender | Female - Male | -0.02 | 0.008 | -0.03 | 0.00 |
| Age | 55 years -⁠ 35 years | -0.04 | <0.001 | -0.05 | -0.03 |
| Ethnicity | Turkish - German | 0.01 | 0.141 | 0.00 | 0.02 |
| Work quality | High - Medium | -0.18 | <0.001 | -0.19 | -0.16 |
| Work quantity | High - Medium | -0.13 | <0.001 | -0.14 | -0.11 |
| Social skills | High - Medium | -0.14 | <0.001 | -0.15 | -0.13 |
| **Layoff expectations** | |  |  |  |  |
| Vaccination | Vaccination refusal - Fully vaccinated | 0.20 | <0.001 | 0.19 | 0.22 |
| Gender | Female - Male | 0.02 | <0.001 | 0.01 | 0.03 |
| Age | 55 years -⁠ 35 years | 0.05 | <0.001 | 0.04 | 0.07 |
| Ethnicity | Turkish - German | 0.08 | <0.001 | 0.06 | 0.09 |
| Work quality | High - Medium | -0.10 | <0.001 | -0.11 | -0.09 |
| Work quantity | High - Medium | -0.07 | <0.001 | -0.08 | -0.06 |
| Social skills | High - Medium | -0.07 | <0.001 | -0.09 | -0.06 |

***Notes:*** *Δ P(.)* represents the average contrast between levels of experimental factors, i.e., the difference in predicted average probabilities between both levels of experimental factors. *p* is the *p-*value adjusted by the false discovery rate. *95%-CI* represents 95% confidence interval. Estimates from Models 1a and 1b in Supplement Tables S7 and S8.

**Table S4: Difference-in-differences of vaccination refusal contrasts by ascribed status group (*N*=12,136 individuals).**

|  |  |  | *95%-CI* | |
| --- | --- | --- | --- | --- |
| Status group | *Diff-in-diff* | *p* | Lower | Upper |
| **Layoff preferences** |  |  |  |  |
| Female - Male | 0.03 | 0.289 | -0.02 | 0.08 |
| 55 years - 35 years | 0.02 | 0.464 | -0.03 | 0.07 |
| Turkish - German | -0.01 | 0.652 | -0.06 | 0.04 |
| **Layoff expectations** |  |  |  |  |
| Female - Male | 0.11 | <0.001 | 0.06 | 0.15 |
| 55 years - 35 years | 0.12 | <0.001 | 0.07 | 0.17 |
| Turkish - German | -0.08 | 0.002 | -0.12 | -0.03 |

***Notes:*** *Diff-in-diff* (difference in difference) represents the differential effect of vaccination refusal depending on ascribed status group, e.g., the difference in the predicted average probabilities of refusing vaccination between females and males. *p* is the *p-*value adjusted by the false discovery rate. *95%-CI* represents 95% confidence interval. Estimates from Models 4a and 4b in Supplement Tables S7 and S8.

**Table S5: Difference-in-differences of vaccination refusal contrasts by work performance (*N*=12,136 individuals).**

|  |  |  | *95%-CI* | |
| --- | --- | --- | --- | --- |
| Performance | *Diff-in-diff* | *p* | Lower | Upper |
| **Layoff preferences** |  |  |  |  |
| Work quality (High - Medium) | -0.07 | 0.009 | -0.12 | -0.02 |
| Work quantity (High - Medium) | 0.04 | 0.179 | -0.01 | 0.09 |
| Social skills (High - Medium) | -0.11 | <0.001 | -0.16 | -0.06 |
| **Layoff expectations** |  |  |  |  |
| Work quality (High - Medium) | 0.04 | 0.145 | -0.01 | 0.08 |
| Work quantity (High - Medium) | -0.01 | 0.703 | -0.06 | 0.04 |
| Social skills (High - Medium) | -0.19 | <0.001 | -0.24 | -0.14 |

***Notes:*** *Diff-in-diff* (difference in difference) represents the differential effect of vaccination refusal between levels of work performance indicators, e.g., the difference in the predicted average probabilities of refusing vaccination between candidates with high and medium work quality. *p* is the *p-*value adjusted by the false discovery rate. *95%-CI* represents 95% confidence interval. Estimates from Models 4a and 4b in Supplement Tables S7 and S8.

**Table S6: Difference-in-differences-in-differences of vaccination refusal contrasts by ascribed status group and work performance (*N*=12,136 individuals).**

|  |  |  |  | *95%-CI* | |
| --- | --- | --- | --- | --- | --- |
| Characteristic | Performance | *Diff-in-diff-in-diff* | *p* | Lower | Upper |
| **Layoff preferences** | | | | | |
| Female - Male | Work quality (High - Medium) | -0.07 | 0.012 | -0.12 | -0.02 |
| Female - Male | Work quantity (High - Medium) | -0.05 | 0.060 | -0.10 | 0.00 |
| Female - Male | Social skills (High - Medium) | -0.10 | <0.001 | -0.16 | -0.05 |
| 55 years - 35 years | Work quality (High - Medium) | -0.01 | 0.797 | -0.06 | 0.04 |
| 55 years - 35 years | Work quantity (High - Medium) | 0.05 | 0.060 | 0.00 | 0.10 |
| 55 years - 35 years | Social skills (High - Medium) | -0.04 | 0.127 | -0.10 | 0.01 |
| Turkish - German | Work quality (High - Medium) | 0.08 | 0.002 | 0.03 | 0.13 |
| Turkish - German | Work quantity (High - Medium) | 0.01 | 0.663 | -0.04 | 0.06 |
| Turkish - German | Social skills (High - Medium) | 0.05 | 0.120 | -0.01 | 0.10 |
| **Layoff expectations** | | | | | |
| Female - Male | Work quality (High - Medium) | 0.03 | 0.305 | -0.02 | 0.08 |
| Female - Male | Work quantity (High - Medium) | 0.00 | 0.923 | -0.05 | 0.05 |
| Female - Male | Social skills (High - Medium) | -0.18 | <0.001 | -0.23 | -0.13 |
| 55 years - 35 years | Work quality (High - Medium) | 0.12 | <0.001 | 0.08 | 0.17 |
| 55 years - 35 years | Work quantity (High - Medium) | -0.03 | 0.268 | -0.08 | 0.02 |
| 55 years - 35 years | Social skills (High - Medium) | -0.06 | 0.025 | -0.11 | -0.01 |
| Turkish - German | Work quality (High - Medium) | 0.01 | 0.808 | -0.04 | 0.05 |
| Turkish - German | Work quantity (High - Medium) | -0.09 | <0.001 | -0.14 | -0.04 |
| Turkish - German | Social skills (High - Medium) | 0.15 | <0.001 | 0.11 | 0.20 |

***Notes:*** *Diff-in-diff-in-diff* (difference in difference in difference) represents the differential effect of vaccination refusal between ascribed status groups depending on work performance indicators. *p* is the *p-*value adjusted by the false discovery rate. *95%-CI* represents 95% confidence interval. Estimates from Models 4a and 4b in Supplement Tables S7 and S8.

**Table S7: Mixed logit models predicting layoff preferences.**

|  | **M1a** | | | **M2a** | | | **M3a** | | | **M4a** | | | **M5a** | | |
| --- | --- | --- | --- | --- | --- | --- | --- | --- | --- | --- | --- | --- | --- | --- | --- |
| *Predictors* | *OR* | *95%-CI* | *p* | *OR* | *95%-CI* | *p* | *ORs* | *95%-CI* | *p* | *OR* | *95%-CI* | *p* | *OR* | *95%-CI* | *p* |
| Intercept | 1.85 | 1.71 – 1.99 | **<0.001** | 1.84 | 1.68 – 2.02 | **<0.001** | 1.86 | 1.68 – 2.06 | **<0.001** | 1.73 | 1.49 – 2.01 | **<0.001** | 1.15 | 1.05 – 1.27 | **0.003** |
| Vaccination refusal (Fully vaccinated) | 2.62 | 2.48 – 2.77 | **<0.001** | 2.63 | 2.36 – 2.92 | **<0.001** | 2.58 | 2.23 – 2.99 | **<0.001** | 2.49 | 1.99 – 3.12 | **<0.001** | 7.34 | 6.66 – 8.10 | **<0.001** |
| Female (Male) | 0.93 | 0.88 – 0.98 | **0.008** | 0.93 | 0.86 – 1.00 | 0.057 | 0.93 | 0.86 – 1.00 | 0.062 | 0.82 | 0.71 – 0.96 | **0.013** | 0.93 | 0.88 – 0.98 | **0.008** |
| 55 years (35 years) | 0.83 | 0.79 – 0.88 | **<0.001** | 0.79 | 0.73 – 0.85 | **<0.001** | 0.79 | 0.73 – 0.85 | **<0.001** | 0.77 | 0.66 – 0.90 | **0.001** | 0.82 | 0.78 – 0.86 | **<0.001** |
| Turkish ethnicity (German) | 1.04 | 0.99 – 1.10 | 0.141 | 1.10 | 1.02 – 1.18 | **0.015** | 1.10 | 1.02 – 1.19 | **0.012** | 1.47 | 1.26 – 1.71 | **<0.001** | 1.04 | 0.99 – 1.10 | 0.129 |
| High work quality (Medium) | 0.46 | 0.43 – 0.48 | **<0.001** | 0.46 | 0.43 – 0.48 | **<0.001** | 0.50 | 0.46 – 0.53 | **<0.001** | 0.57 | 0.49 – 0.67 | **<0.001** | 0.45 | 0.42 – 0.47 | **<0.001** |
| High work quantity (Medium) | 0.57 | 0.54 – 0.60 | **<0.001** | 0.57 | 0.54 – 0.60 | **<0.001** | 0.51 | 0.47 – 0.55 | **<0.001** | 0.63 | 0.54 – 0.74 | **<0.001** | 0.56 | 0.53 – 0.59 | **<0.001** |
| High social skills (Medium) | 0.54 | 0.51 – 0.57 | **<0.001** | 0.54 | 0.51 – 0.57 | **<0.001** | 0.54 | 0.50 – 0.58 | **<0.001** | 0.44 | 0.38 – 0.51 | **<0.001** | 0.53 | 0.50 – 0.56 | **<0.001** |
| Vaccination refusal * Female |  |  |  | 1.00 | 0.90 – 1.11 | 0.988 | 1.00 | 0.90 – 1.11 | 0.988 | 1.65 | 1.31 – 2.07 | **<0.001** |  |  |  |
| Vaccination refusal * Age 55 |  |  |  | 1.11 | 1.00 – 1.24 | 0.051 | 1.11 | 1.00 – 1.24 | 0.056 | 1.00 | 0.80 – 1.26 | 0.976 |  |  |  |
| Vaccination refusal * Turkish ethnicity |  |  |  | 0.90 | 0.80 – 1.00 | **0.044** | 0.89 | 0.80 – 1.00 | **0.041** | 0.67 | 0.53 – 0.84 | **<0.001** |  |  |  |
| Vaccination refusal * High work quality |  |  |  |  |  |  | 0.85 | 0.76 – 0.94 | **0.003** | 0.82 | 0.66 – 1.02 | 0.072 |  |  |  |
| Vaccination refusal * High work quantity |  |  |  |  |  |  | 1.25 | 1.12 – 1.39 | **<0.001** | 1.11 | 0.90 – 1.38 | 0.339 |  |  |  |
| Vaccination refusal * High social skills |  |  |  |  |  |  | 0.99 | 0.89 – 1.10 | 0.873 | 1.21 | 0.97 – 1.50 | 0.092 |  |  |  |
| Female * High work quality |  |  |  |  |  |  |  |  |  | 1.02 | 0.88 – 1.19 | 0.762 |  |  |  |
| Age 55 * High work quality |  |  |  |  |  |  |  |  |  | 0.92 | 0.79 – 1.07 | 0.277 |  |  |  |
| Turkish ethnicity * High work quality |  |  |  |  |  |  |  |  |  | 0.79 | 0.68 – 0.92 | **0.003** |  |  |  |
| Female * High work quantity |  |  |  |  |  |  |  |  |  | 0.95 | 0.81 – 1.11 | 0.506 |  |  |  |
| Age 55 * High work quantity |  |  |  |  |  |  |  |  |  | 0.88 | 0.76 – 1.03 | 0.106 |  |  |  |
| Turkish ethnicity * High work quantity |  |  |  |  |  |  |  |  |  | 0.77 | 0.66 – 0.90 | **0.001** |  |  |  |
| Female * High social skills |  |  |  |  |  |  |  |  |  | 1.32 | 1.13 – 1.54 | **<0.001** |  |  |  |
| Age 55 * High social skills |  |  |  |  |  |  |  |  |  | 1.29 | 1.11 – 1.50 | **0.001** |  |  |  |
| Turkish ethnicity * High social skills |  |  |  |  |  |  |  |  |  | 0.90 | 0.77 – 1.05 | 0.170 |  |  |  |
| Vaccination refusal * Female * High work quality |  |  |  |  |  |  |  |  |  | 0.69 | 0.55 – 0.86 | **0.001** |  |  |  |
| Vaccination refusal * Age 55 * High work quality |  |  |  |  |  |  |  |  |  | 1.08 | 0.87 – 1.34 | 0.505 |  |  |  |
| Vaccination refusal * Turkish ethnicity * High work quality |  |  |  |  |  |  |  |  |  | 1.41 | 1.14 – 1.76 | **0.002** |  |  |  |
| Vaccination refusal * Female * High work quantity |  |  |  |  |  |  |  |  |  | 0.85 | 0.68 – 1.05 | 0.131 |  |  |  |
| Vaccination refusal * Age 55 * High work quantity |  |  |  |  |  |  |  |  |  | 1.36 | 1.09 – 1.69 | **0.006** |  |  |  |
| Vaccination refusal * Turkish ethnicity * High work quantity |  |  |  |  |  |  |  |  |  | 1.09 | 0.88 – 1.36 | 0.429 |  |  |  |
| Vaccination refusal * Female * High social skills |  |  |  |  |  |  |  |  |  | 0.64 | 0.52 – 0.80 | **<0.001** |  |  |  |
| Vaccination refusal * Age 55 * High social skills |  |  |  |  |  |  |  |  |  | 0.85 | 0.68 – 1.06 | 0.148 |  |  |  |
| Vaccination refusal * Turkish ethnicity * High social skills |  |  |  |  |  |  |  |  |  | 1.20 | 0.96 – 1.49 | 0.104 |  |  |  |
| Negative attitude toward vaccination |  |  |  |  |  |  |  |  |  |  |  |  | 1.30 | 1.26 – 1.33 | **<0.001** |
| Vaccination refusal * Negative attitude toward vaccination |  |  |  |  |  |  |  |  |  |  |  |  | 0.59 | 0.57 – 0.62 | **<0.001** |
| *N*_Individuals_ | 12,136 | | | 12,136 | | | 12,136 | | | 12,136 | | | 12,029 | | |
| *N*_Observations_ | 24,272 | | | 24,272 | | | 24,272 | | | 24,272 | | | 24,058 | | |
| *Marginal* *R*² | 0.147 | | | 0.147 | | | 0.149 | | | 0.153 | | | 0.179 | | |

***Notes:*** *OR* represents Odds Ratios. *95%-CI* represents 95% confidence interval.

**Table S8: Mixed logit models predicting layoff expectations.**

|  | **M1b** | | | **M2b** | | | **M3b** | | | **M4b** | | | **M5b** | | |
| --- | --- | --- | --- | --- | --- | --- | --- | --- | --- | --- | --- | --- | --- | --- | --- |
| *Predictors* | *OR* | *95%-CI* | *p* | *OR* | *95%-CI* | *p* | *OR* | *95%-CI* | *p* | *OR* | *95%-CI* | *p* | *OR* | *95%-CI* | *p* |
| Intercept | 0.80 | 0.75 – 0.87 | **<0.001** | 0.79 | 0.73 – 0.87 | **<0.001** | 0.73 | 0.66 – 0.80 | **<0.001** | 0.67 | 0.58 – 0.78 | **<0.001** | 0.80 | 0.73 – 0.88 | **<0.001** |
| Vaccination refusal (Fully vaccinated) | 2.35 | 2.23 – 2.48 | **<0.001** | 2.39 | 2.15 – 2.66 | **<0.001** | 2.85 | 2.48 – 3.28 | **<0.001** | 2.49 | 2.01 – 3.09 | **<0.001** | 2.41 | 2.20 – 2.65 | **<0.001** |
| Female (Male) | 1.10 | 1.04 – 1.15 | **0.001** | 1.06 | 0.99 – 1.14 | 0.106 | 1.06 | 0.99 – 1.15 | 0.094 | 1.09 | 0.94 – 1.26 | 0.272 | 1.10 | 1.04 – 1.16 | **0.001** |
| 55 years (35 years) | 1.26 | 1.20 – 1.33 | **<0.001** | 1.20 | 1.12 – 1.29 | **<0.001** | 1.20 | 1.11 – 1.29 | **<0.001** | 1.24 | 1.07 – 1.44 | **0.004** | 1.26 | 1.20 – 1.33 | **<0.001** |
| Turkish ethnicity (German) | 1.38 | 1.31 – 1.45 | **<0.001** | 1.52 | 1.41 – 1.63 | **<0.001** | 1.52 | 1.41 – 1.64 | **<0.001** | 1.70 | 1.47 – 1.98 | **<0.001** | 1.38 | 1.31 – 1.45 | **<0.001** |
| High work quality (Medium) | 0.65 | 0.61 – 0.68 | **<0.001** | 0.65 | 0.61 – 0.68 | **<0.001** | 0.73 | 0.67 – 0.78 | **<0.001** | 0.96 | 0.82 – 1.12 | 0.604 | 0.64 | 0.61 – 0.68 | **<0.001** |
| High work quantity (Medium) | 0.74 | 0.70 – 0.78 | **<0.001** | 0.74 | 0.70 – 0.78 | **<0.001** | 0.81 | 0.75 – 0.87 | **<0.001** | 0.88 | 0.76 – 1.03 | 0.109 | 0.74 | 0.70 – 0.78 | **<0.001** |
| High social skills (Medium) | 0.73 | 0.69 – 0.76 | **<0.001** | 0.73 | 0.69 – 0.76 | **<0.001** | 0.71 | 0.66 – 0.76 | **<0.001** | 0.56 | 0.48 – 0.65 | **<0.001** | 0.73 | 0.69 – 0.77 | **<0.001** |
| Vaccination refusal * Female |  |  |  | 1.06 | 0.96 – 1.18 | 0.249 | 1.06 | 0.96 – 1.18 | 0.253 | 1.56 | 1.25 – 1.94 | **<0.001** |  |  |  |
| Vaccination refusal * Age 55 |  |  |  | 1.10 | 0.99 – 1.22 | 0.067 | 1.10 | 0.99 – 1.23 | 0.065 | 1.07 | 0.86 – 1.33 | 0.540 |  |  |  |
| Vaccination refusal * Turkish ethnicity |  |  |  | 0.82 | 0.74 – 0.91 | **<0.001** | 0.83 | 0.74 – 0.92 | **<0.001** | 0.81 | 0.65 – 1.00 | 0.052 |  |  |  |
| Vaccination refusal * High work quality |  |  |  |  |  |  | 0.80 | 0.72 – 0.89 | **<0.001** | 0.64 | 0.52 – 0.79 | **<0.001** |  |  |  |
| Vaccination refusal * High work quantity |  |  |  |  |  |  | 0.84 | 0.76 – 0.93 | **0.001** | 1.11 | 0.90 – 1.37 | 0.332 |  |  |  |
| Vaccination refusal * High social skills |  |  |  |  |  |  | 1.05 | 0.94 – 1.16 | 0.371 | 1.29 | 1.05 – 1.60 | **0.018** |  |  |  |
| Female * High work quality |  |  |  |  |  |  |  |  |  | 0.90 | 0.78 – 1.05 | 0.182 |  |  |  |
| Age 55 * High work quality |  |  |  |  |  |  |  |  |  | 0.68 | 0.59 – 0.79 | **<0.001** |  |  |  |
| Turkish ethnicity * High work quality |  |  |  |  |  |  |  |  |  | 0.92 | 0.79 – 1.07 | 0.267 |  |  |  |
| Female * High work quantity |  |  |  |  |  |  |  |  |  | 0.76 | 0.66 – 0.89 | **<0.001** |  |  |  |
| Age 55 * High work quantity |  |  |  |  |  |  |  |  |  | 0.92 | 0.79 – 1.06 | 0.244 |  |  |  |
| Turkish ethnicity * High work quantity |  |  |  |  |  |  |  |  |  | 1.17 | 1.00 – 1.35 | **0.044** |  |  |  |
| Female * High social skills |  |  |  |  |  |  |  |  |  | 1.39 | 1.20 – 1.61 | **<0.001** |  |  |  |
| Age 55 * High social skills |  |  |  |  |  |  |  |  |  | 1.56 | 1.34 – 1.81 | **<0.001** |  |  |  |
| Turkish ethnicity * High social skills |  |  |  |  |  |  |  |  |  | 0.74 | 0.64 – 0.86 | **<0.001** |  |  |  |
| Vaccination refusal * Female * High work quality |  |  |  |  |  |  |  |  |  | 1.03 | 0.83 – 1.27 | 0.783 |  |  |  |
| Vaccination refusal * Age 55 * High work quality |  |  |  |  |  |  |  |  |  | 1.59 | 1.29 – 1.97 | **<0.001** |  |  |  |
| Vaccination refusal * Turkish ethnicity * High work quality |  |  |  |  |  |  |  |  |  | 0.92 | 0.74 – 1.13 | 0.428 |  |  |  |
| Vaccination refusal * Female * High work quantity |  |  |  |  |  |  |  |  |  | 1.01 | 0.81 – 1.24 | 0.957 |  |  |  |
| Vaccination refusal * Age 55 * High work quantity |  |  |  |  |  |  |  |  |  | 0.89 | 0.72 – 1.10 | 0.278 |  |  |  |
| Vaccination refusal * Turkish ethnicity * High work quantity |  |  |  |  |  |  |  |  |  | 0.64 | 0.52 – 0.79 | **<0.001** |  |  |  |
| Vaccination refusal * Female * High social skills |  |  |  |  |  |  |  |  |  | 0.46 | 0.37 – 0.57 | **<0.001** |  |  |  |
| Vaccination refusal * Age 55 * High social skills |  |  |  |  |  |  |  |  |  | 0.73 | 0.59 – 0.90 | **0.004** |  |  |  |
| Vaccination refusal * Turkish ethnicity * High social skills |  |  |  |  |  |  |  |  |  | 1.88 | 1.52 – 2.32 | **<0.001** |  |  |  |
| Negative attitude toward vaccination |  |  |  |  |  |  |  |  |  |  |  |  | 1.00 | 0.98 – 1.03 | 0.817 |
| Vaccination refusal * Negative attitude toward vaccination |  |  |  |  |  |  |  |  |  |  |  |  | 0.99 | 0.95 – 1.03 | 0.459 |
| *N*_Individuals_ | 12,136 | | | 12,136 | | | 12,136 | | | 12,136 | | | 12,029 | | |
| *N*_Observations_ | 24,272 | | | 24,272 | | | 24,272 | | | 24,272 | | | 24,058 | | |
| *Marginal* *R*^2^ | 0.089 | | | 0.090 | | | 0.092 | | | 0.102 | | | 0.089 | | |

***Notes:*** *OR* represents Odds Ratios. *95%-CI* represents 95% confidence interval.

**Table S9: Mixed logit models predicting layoff preferences. Robustness tests with all dimensions.**

|  | **M1a** | | | **M2a** | | | **M3a** | | | **M4a** | | |
| --- | --- | --- | --- | --- | --- | --- | --- | --- | --- | --- | --- | --- |
| *Predictors* | *OR* | *95%-CI* | *p* | *OR* | *95%-CI* | *p* | *OR* | *95%-CI* | *p* | *OR* | *95%-CI* | *p* |
| Intercept | 2.06 | 1.89 – 2.25 | **<0.001** | 2.11 | 1.91 – 2.33 | **<0.001** | 2.11 | 1.89 – 2.35 | **<0.001** | 1.98 | 1.69 – 2.32 | **<0.001** |
| Vaccination refusal (Fully vaccinated) | 2.69 | 2.54 – 2.84 | **<0.001** | 2.58 | 2.31 – 2.88 | **<0.001** | 2.62 | 2.26 – 3.04 | **<0.001** | 2.46 | 1.96 – 3.08 | **<0.001** |
| Female (Male) | 0.93 | 0.88 – 0.98 | **0.008** | 0.93 | 0.87 – 1.01 | 0.085 | 0.94 | 0.87 – 1.01 | 0.094 | 0.82 | 0.70 – 0.96 | **0.013** |
| Turkish ethnicity (German) | 1.04 | 0.99 – 1.10 | 0.133 | 1.11 | 1.02 – 1.19 | **0.010** | 1.11 | 1.03 – 1.20 | **0.009** | 1.52 | 1.30 – 1.78 | **<0.001** |
| 55 years (35 years) | 0.83 | 0.79 – 0.88 | **<0.001** | 0.74 | 0.69 – 0.80 | **<0.001** | 0.75 | 0.69 – 0.81 | **<0.001** | 0.69 | 0.59 – 0.81 | **<0.001** |
| Tenure (2 years) | 0.57 | 0.54 – 0.60 | **<0.001** | 0.56 | 0.53 – 0.60 | **<0.001** | 0.56 | 0.53 – 0.59 | **<0.001** | 0.56 | 0.53 – 0.59 | **<0.001** |
| High work quality (Medium) | 0.45 | 0.42 – 0.47 | **<0.001** | 0.45 | 0.43 – 0.47 | **<0.001** | 0.49 | 0.45 – 0.53 | **<0.001** | 0.58 | 0.49 – 0.67 | **<0.001** |
| High work quantity (Medium) | 0.57 | 0.54 – 0.60 | **<0.001** | 0.57 | 0.54 – 0.60 | **<0.001** | 0.51 | 0.47 – 0.55 | **<0.001** | 0.61 | 0.52 – 0.72 | **<0.001** |
| High social skills (Medium) | 0.53 | 0.50 – 0.56 | **<0.001** | 0.53 | 0.50 – 0.56 | **<0.001** | 0.55 | 0.51 – 0.59 | **<0.001** | 0.44 | 0.38 – 0.52 | **<0.001** |
| Nepotism (none) | 1.44 | 1.37 – 1.52 | **<0.001** | 1.45 | 1.37 – 1.53 | **<0.001** | 1.44 | 1.37 – 1.52 | **<0.001** | 1.46 | 1.38 – 1.54 | **<0.001** |
| Vaccination refusal * Female |  |  |  | 0.98 | 0.88 – 1.10 | 0.755 | 0.98 | 0.88 – 1.09 | 0.743 | 1.65 | 1.31 – 2.08 | **<0.001** |
| Vaccination refusal * Age 55 |  |  |  | 1.25 | 1.12 – 1.39 | **<0.001** | 1.24 | 1.12 – 1.39 | **<0.001** | 1.16 | 0.92 – 1.46 | 0.220 |
| Vaccination refusal * Turkish ethnicity |  |  |  | 0.88 | 0.79 – 0.99 | **0.027** | 0.88 | 0.79 – 0.99 | **0.026** | 0.68 | 0.54 – 0.86 | **0.001** |
| Vaccination refusal * High work quality |  |  |  |  |  |  | 0.84 | 0.75 – 0.93 | **0.001** | 0.79 | 0.63 – 0.98 | **0.032** |
| Vaccination refusal * High work quantity |  |  |  |  |  |  | 1.25 | 1.12 – 1.39 | **<0.001** | 1.16 | 0.93 – 1.45 | 0.178 |
| Vaccination refusal * High social skills |  |  |  |  |  |  | 0.94 | 0.84 – 1.05 | 0.277 | 1.19 | 0.95 – 1.48 | 0.122 |
| Female * High work quality |  |  |  |  |  |  |  |  |  | 1.02 | 0.87 – 1.19 | 0.804 |
| Age 55 * High work quality |  |  |  |  |  |  |  |  |  | 0.95 | 0.82 – 1.11 | 0.557 |
| Turkish ethnicity * High work quality |  |  |  |  |  |  |  |  |  | 0.74 | 0.63 – 0.86 | **<0.001** |
| Female * High work quantity |  |  |  |  |  |  |  |  |  | 0.95 | 0.82 – 1.11 | 0.528 |
| Age 55 * High work quantity |  |  |  |  |  |  |  |  |  | 0.95 | 0.81 – 1.11 | 0.493 |
| Turkish ethnicity * High work quantity |  |  |  |  |  |  |  |  |  | 0.78 | 0.66 – 0.91 | **0.001** |
| Female * High social skills |  |  |  |  |  |  |  |  |  | 1.33 | 1.14 – 1.56 | **<0.001** |
| Age 55 * High social skills |  |  |  |  |  |  |  |  |  | 1.30 | 1.11 – 1.52 | **0.001** |
| Turkish ethnicity * High social skills |  |  |  |  |  |  |  |  |  | 0.90 | 0.77 – 1.05 | 0.195 |
| Vaccination refusal * Female * High work quality |  |  |  |  |  |  |  |  |  | 0.72 | 0.57 – 0.89 | **0.003** |
| Vaccination refusal * Age 55 * High work quality |  |  |  |  |  |  |  |  |  | 1.07 | 0.86 – 1.34 | 0.547 |
| Vaccination refusal * Turkish ethnicity * High work quality |  |  |  |  |  |  |  |  |  | 1.44 | 1.15 – 1.79 | **0.001** |
| Vaccination refusal * Female * High work quantity |  |  |  |  |  |  |  |  |  | 0.83 | 0.66 – 1.03 | 0.092 |
| Vaccination refusal * Age 55 * High work quantity |  |  |  |  |  |  |  |  |  | 1.33 | 1.07 – 1.66 | **0.011** |
| Vaccination refusal * Turkish ethnicity * High work quantity |  |  |  |  |  |  |  |  |  | 1.03 | 0.83 – 1.28 | 0.798 |
| Vaccination refusal * Female * High social skills |  |  |  |  |  |  |  |  |  | 0.61 | 0.49 – 0.76 | **<0.001** |
| Vaccination refusal * Age 55 * High social skills |  |  |  |  |  |  |  |  |  | 0.84 | 0.67 – 1.04 | 0.113 |
| Vaccination refusal * Turkish ethnicity * High social skills |  |  |  |  |  |  |  |  |  | 1.17 | 0.94 – 1.46 | 0.158 |
| *N*_Individuals_ | 12,136 | | | 12,136 | | | 12,136 | | | 12,136 | | |
| *N*_Observations_ | 24,272 | | | 24,272 | | | 24,272 | | | 24,272 | | |
| *Marginal* *R*^2^ | 0.176 | | | 0.177 | | | 0.179 | | | 0.183 | | |

***Notes:*** *OR* represents Odds Ratios. *95%-CI* represents 95% confidence interval.

**Table S10: Mixed logit models predicting layoff expectations. Robustness tests with all dimensions.**

|  | **M1b** | | | **M2b** | | | **M3b** | | | **M4b** | | |
| --- | --- | --- | --- | --- | --- | --- | --- | --- | --- | --- | --- | --- |
| *Predictors* | *OR* | *95%-CI* | *p* | *OR* | *95%-CI* | *p* | *OR* | *95%-CI* | *p* | *OR* | *95%-CI* | *p* |
| Intercept | 1.84 | 1.69 – 2.01 | **<0.001** | 1.77 | 1.60 – 1.96 | **<0.001** | 1.66 | 1.49 – 1.86 | **<0.001** | 1.61 | 1.37 – 1.89 | **<0.001** |
| Vaccination refusal (Fully vaccinated) | 2.58 | 2.44 – 2.73 | **<0.001** | 2.80 | 2.50 – 3.13 | **<0.001** | 3.19 | 2.76 – 3.70 | **<0.001** | 2.71 | 2.17 – 3.39 | **<0.001** |
| Female (Male) | 1.10 | 1.04 – 1.16 | **0.001** | 1.04 | 0.96 – 1.13 | 0.294 | 1.04 | 0.97 – 1.13 | 0.276 | 0.96 | 0.82 – 1.13 | 0.648 |
| Turkish ethnicity (German) | 1.43 | 1.35 – 1.51 | **<0.001** | 1.67 | 1.54 – 1.80 | **<0.001** | 1.67 | 1.55 – 1.81 | **<0.001** | 1.85 | 1.57 – 2.16 | **<0.001** |
| 55 years (35 years) | 1.32 | 1.25 – 1.40 | **<0.001** | 1.30 | 1.21 – 1.41 | **<0.001** | 1.30 | 1.20 – 1.41 | **<0.001** | 1.42 | 1.22 – 1.67 | **<0.001** |
| Tenure (2 years) | 0.67 | 0.64 – 0.71 | **<0.001** | 0.67 | 0.63 – 0.70 | **<0.001** | 0.67 | 0.63 – 0.71 | **<0.001** | 0.66 | 0.63 – 0.70 | **<0.001** |
| High work quality (Medium) | 0.61 | 0.58 – 0.65 | **<0.001** | 0.61 | 0.58 – 0.65 | **<0.001** | 0.71 | 0.66 – 0.77 | **<0.001** | 0.88 | 0.75 – 1.04 | 0.124 |
| High work quantity (Medium) | 0.72 | 0.68 – 0.76 | **<0.001** | 0.72 | 0.68 – 0.76 | **<0.001** | 0.73 | 0.68 – 0.79 | **<0.001** | 0.85 | 0.73 – 1.00 | 0.055 |
| High social skills (Medium) | 0.71 | 0.67 – 0.75 | **<0.001** | 0.70 | 0.67 – 0.74 | **<0.001** | 0.68 | 0.63 – 0.73 | **<0.001** | 0.49 | 0.42 – 0.58 | **<0.001** |
| Nepotism (none) | 0.26 | 0.25 – 0.28 | **<0.001** | 0.26 | 0.25 – 0.28 | **<0.001** | 0.26 | 0.25 – 0.27 | **<0.001** | 0.26 | 0.24 – 0.27 | **<0.001** |
| Vaccination refusal * Female |  |  |  | 1.12 | 1.01 – 1.26 | **0.038** | 1.13 | 1.01 – 1.26 | **0.034** | 1.65 | 1.31 – 2.08 | **<0.001** |
| Vaccination refusal * Age 55 |  |  |  | 1.03 | 0.92 – 1.15 | 0.592 | 1.03 | 0.92 – 1.15 | 0.629 | 0.88 | 0.70 – 1.11 | 0.286 |
| Vaccination refusal * Turkish ethnicity |  |  |  | 0.73 | 0.66 – 0.82 | **<0.001** | 0.73 | 0.66 – 0.82 | **<0.001** | 0.85 | 0.68 – 1.07 | 0.175 |
| Vaccination refusal * High work quality |  |  |  |  |  |  | 0.74 | 0.66 – 0.83 | **<0.001** | 0.62 | 0.50 – 0.77 | **<0.001** |
| Vaccination refusal * High work quantity |  |  |  |  |  |  | 0.96 | 0.86 – 1.08 | 0.518 | 1.10 | 0.88 – 1.37 | 0.416 |
| Vaccination refusal * High social skills |  |  |  |  |  |  | 1.09 | 0.97 – 1.21 | 0.143 | 1.60 | 1.28 – 2.01 | **<0.001** |
| Female * High work quality |  |  |  |  |  |  |  |  |  | 0.92 | 0.79 – 1.08 | 0.331 |
| Age 55 * High work quality |  |  |  |  |  |  |  |  |  | 0.68 | 0.58 – 0.79 | **<0.001** |
| Turkish ethnicity * High work quality |  |  |  |  |  |  |  |  |  | 1.03 | 0.88 – 1.21 | 0.685 |
| Female * High work quantity |  |  |  |  |  |  |  |  |  | 0.80 | 0.69 – 0.94 | **0.006** |
| Age 55 * High work quantity |  |  |  |  |  |  |  |  |  | 0.85 | 0.72 – 0.99 | **0.038** |
| Turkish ethnicity * High work quantity |  |  |  |  |  |  |  |  |  | 1.04 | 0.89 – 1.22 | 0.618 |
| Female * High social skills |  |  |  |  |  |  |  |  |  | 1.61 | 1.37 – 1.88 | **<0.001** |
| Age 55 * High social skills |  |  |  |  |  |  |  |  |  | 1.55 | 1.32 – 1.81 | **<0.001** |
| Turkish ethnicity * High social skills |  |  |  |  |  |  |  |  |  | 0.75 | 0.64 – 0.88 | **<0.001** |
| Vaccination refusal * Female * High work quality |  |  |  |  |  |  |  |  |  | 1.18 | 0.94 – 1.48 | 0.148 |
| Vaccination refusal * Age 55 * High work quality |  |  |  |  |  |  |  |  |  | 1.60 | 1.28 – 2.01 | **<0.001** |
| Vaccination refusal * Turkish ethnicity * High work quality |  |  |  |  |  |  |  |  |  | 0.73 | 0.58 – 0.91 | **0.005** |
| Vaccination refusal * Female * High work quantity |  |  |  |  |  |  |  |  |  | 1.08 | 0.86 – 1.35 | 0.493 |
| Vaccination refusal * Age 55 * High work quantity |  |  |  |  |  |  |  |  |  | 0.98 | 0.78 – 1.22 | 0.841 |
| Vaccination refusal * Turkish ethnicity * High work quantity |  |  |  |  |  |  |  |  |  | 0.73 | 0.59 – 0.92 | **0.006** |
| Vaccination refusal * Female * High social skills |  |  |  |  |  |  |  |  |  | 0.37 | 0.29 – 0.46 | **<0.001** |
| Vaccination refusal * Age 55 * High social skills |  |  |  |  |  |  |  |  |  | 0.85 | 0.68 – 1.06 | 0.142 |
| Vaccination refusal * Turkish ethnicity * High social skills |  |  |  |  |  |  |  |  |  | 1.46 | 1.16 – 1.82 | **0.001** |
| *N*_Individuals_ | 12,136 | | | 12,136 | | | 12,136 | | | 12,136 | | |
| *N*_Observations_ | 24,272 | | | 24,272 | | | 24,272 | | | 24,272 | | |
| *Marginal* *R*^2^ | 0.214 | | | 0.216 | | | 0.218 | | | 0.228 | | |

***Notes:*** *OR* represents Odds Ratios. *95%-CI* represents 95% confidence interval.

**Table S11: Robustness test of question order for layoff preferences.**

|  | **M1** | | | **M2** | | | **M3** | | | **M4** | | |
| --- | --- | --- | --- | --- | --- | --- | --- | --- | --- | --- | --- | --- |
| *Predictors* | *OR* | *95%-CI* | *p* | *Estimate* | *95%-CI* | *p* | *OR* | *95%-CI* | *p* | *Estimate* | *95%-CI* | *p* |
| Intercept | 1.00 | 0.97 – 1.04 | 1.000 | 0.50 | 0.49 – 0.51 | **<0.001** | 1.85 | 1.71 – 2.00 | **<0.001** | 0.64 | 0.62 – 0.65 | **<0.001** |
| Question order (preferences and expectations) | 1.00 | 0.95 – 1.05 | 1.000 | 0.00 | -0.01 – 0.01 | 1.000 | 1.00 | 0.95 – 1.06 | 0.981 | 0.00 | -0.01 – 0.01 | 0.966 |
| Vaccination refusal (Fully vaccinated) |  |  |  |  |  |  | 2.62 | 2.49 – 2.77 | **<0.001** | 0.22 | 0.21 – 0.23 | **<0.001** |
| Female (Male) |  |  |  |  |  |  | 0.93 | 0.88 – 0.98 | **0.008** | -0.02 | -0.03 – -0.00 | **0.009** |
| 55 years (35 years) |  |  |  |  |  |  | 0.83 | 0.79 – 0.88 | **<0.001** | -0.04 | -0.05 – -0.03 | **<0.001** |
| Turkish ethnicity (German) |  |  |  |  |  |  | 1.04 | 0.99 – 1.10 | 0.140 | 0.01 | -0.00 – 0.02 | 0.147 |
| High work quality (Medium) |  |  |  |  |  |  | 0.46 | 0.43 – 0.48 | **<0.001** | -0.18 | -0.19 – -0.17 | **<0.001** |
| High work quantity (Medium) |  |  |  |  |  |  | 0.57 | 0.54 – 0.60 | **<0.001** | -0.13 | -0.14 – -0.11 | **<0.001** |
| High social skills (Medium) |  |  |  |  |  |  | 0.54 | 0.51 – 0.57 | **<0.001** | -0.14 | -0.15 – -0.13 | **<0.001** |
| *N*_Individuals_ | 12135 | | | 12135 | | | 12135 | | | 12135 | | |
| *N*_Observations_ | 24270 | | | 24270 | | | 24270 | | | 24270 | | |
| *Marginal* *R*^2^ | 0.000 | | | 0.000 | | | 0.147 | | | 0.116 | | |

***Notes:*** M1 and M3 are mixed logit models; M2 and M4 are linear probability models. *OR* represents Odds Ratios. *95%-CI* represents 95% confidence interval.

**Table S12: Robustness test of question order for layoff expectations.**

|  | **M1** | | | **M2** | | | **M3** | | | **M4** | | |
| --- | --- | --- | --- | --- | --- | --- | --- | --- | --- | --- | --- | --- |
| *Predictors* | *OR* | *95%-CI* | *p* | *Estimates* | *95%-CI* | *p* | *OR* | *95%-CI* | *p* | *Estimates* | *95%-CI* | *p* |
| Intercept | 1.00 | 0.97 – 1.04 | 1.000 | 0.50 | 0.49 – 0.51 | **<0.001** | 0.80 | 0.74 – 0.87 | **<0.001** | 0.45 | 0.43 – 0.46 | **<0.001** |
| Question order (preferences and expectations) | 1.00 | 0.95 – 1.05 | 1.000 | 0.00 | -0.01 – 0.01 | 1.000 | 1.00 | 0.95 – 1.06 | 0.908 | 0.00 | -0.01 – 0.01 | 0.914 |
| Vaccination refusal (Fully vaccinated) |  |  |  |  |  |  | 2.35 | 2.23 – 2.48 | **<0.001** | 0.20 | 0.19 – 0.22 | **<0.001** |
| Female (Male) |  |  |  |  |  |  | 1.10 | 1.04 – 1.15 | **0.001** | 0.02 | 0.01 – 0.03 | **<0.001** |
| 55 years (35 years) |  |  |  |  |  |  | 1.26 | 1.19 – 1.33 | **<0.001** | 0.05 | 0.04 – 0.07 | **<0.001** |
| Turkish ethnicity (German) |  |  |  |  |  |  | 1.38 | 1.31 – 1.45 | **<0.001** | 0.08 | 0.06 – 0.09 | **<0.001** |
| High work quality (Medium) |  |  |  |  |  |  | 0.65 | 0.61 – 0.68 | **<0.001** | -0.10 | -0.11 – -0.09 | **<0.001** |
| High work quantity (Medium) |  |  |  |  |  |  | 0.74 | 0.70 – 0.78 | **<0.001** | -0.07 | -0.08 – -0.06 | **<0.001** |
| High social skills (Medium) |  |  |  |  |  |  | 0.73 | 0.69 – 0.76 | **<0.001** | -0.07 | -0.09 – -0.06 | **<0.001** |
| *N*_Individuals_ | 12,135 | | | 12,135 | | | 12,135 | | | 12,135 | | |
| *N*_Observations_ | 24,270 | | | 24,270 | | | 24,270 | | | 24,270 | | |
| *Marginal* *R*^2^ | 0.000 | | | 0.000 | | | 0.089 | | | 0.072 | | |

***Notes:*** M1 and M3 are mixed logit models; M2 and M4 are linear probability models. *OR* represents Odds Ratios. *95%-CI* represents 95% confidence interval.
